# APP and DNA damage create a feedforward loop that contributes to Alzheimer's disease pathogenesis

**DOI:** 10.1002/alz70855_103872

**Published:** 2025-12-24

**Authors:** Fulin Ma, Rody Kingston, Eliana Brenner, Karl Herrup

**Affiliations:** ^1^ University of Pittsburgh, Pittsburgh, PA, USA; ^2^ University of Pittsburgh School of Medicine, Pittsburgh, PA, USA

## Abstract

**Background:**

Alzheimer's disease (AD) has a typical age of onset exceeding 65 years. The amyloid precursor protein (APP) has occupied a central position in the pathophysiological description of AD. Unfortunately, Aβ‐independent roles for the full‐length APP protein in AD are relatively understudied. Our work begins from the perspective that APP is a damage‐response protein that is elevated after a variety of different stresses.

**Method:**

Immunocytochemistry was performed on mouse brain or on dissociated cultures of neurons. We tracked DNA damage with 53BP1, 𝛾‐H2AX, or the TUNEL reaction. Lentiviruses over expressing human APP is also used.

**Result:**

We demonstrate that neuronal excitation in vitro leads not only to DNA damage but also to a rapid increase in APP message and protein. Induction of DNA damage alone is sufficient to induce APP expression. This can be shown either by directly inducing DNA damage with etoposide in vitro or indirectly by inhibiting DNA repair – a prominent phenotype of the *Atm^‐/^^‐^* mouse. We find that an increase in APP is sufficient in and of itself to cause DNA damage. In APP overexpressing AD mouse models DNA damage is significantly increased. In vitro, infection of cultures of wild type neurons with lentiviruses that overexpress APP also led to significant DNA damage. We applied a BACE1 inhibitor (verbecestat) or a γ‐secretase inhibitor L685458 to block the production of Aβ. The results appeared to support a key role for Aβ since both inhibitors completely blocked the APP‐induced DNA damage response. However, application of oligomeric Aβ directly to neurons resulted in no significant increase in DNA damage. This led to the realization that verubecestat and L685458 would also both inhibit the production of the APP intracellular domain (AICD).

**Conclusion:**

The findings offer a potential linkage between DNA damage, a hallmark of aging, and APP, a hallmark of AD. The linkage between APP and DNA damage appears to be bi‐directional and paints the picture of a pathway with the potential to become a dangerous feed‐forward loop. Our findings reveal a novel role for full length APP in AD pathogenesis through its relationship with DNA damage.

